# Like a rolling stone: Psychotherapy without (episodic) memory

**DOI:** 10.3389/fpsyt.2022.958194

**Published:** 2022-11-02

**Authors:** Paul A. Moore, Oliver Hugh Turnbull

**Affiliations:** ^1^Department of Psychiatry, School of Medicine, Trinity College Dublin, Dublin, Ireland; ^2^School of Human and Behavioral Sciences, Bangor University, Wales, United Kingdom

**Keywords:** amnesia, memory, psychotherapy, psychoanalysis, therapeutic alliance, transference, brain injury

## Abstract

People with profound amnesia still retain the capacity to learn about the *emotional* value of experiences, which is crucial in developing and sustaining interpersonal relationships. In a 2017 paper, we demonstrated for the first time (with patient JL) that transferential feelings develop *across* the therapeutic process, despite profound episodic memory impairment after medial temporal lesions. This paper reports a second case (GA) of a profoundly amnesic patient in psychotherapy, this time after lesions to the anterior fornix. The work with GA opens issues such as the differences and similarities to the previous case, counter-transference phenomena, and the effects of hyperphagia. The findings make it clear that many phenomena are common to both GA and JL, such as forgetfulness, various types of repetition, the importance of the therapeutic alliance, and the ability to make therapeutic gain. However, there were differences between the cases, for example as regards confabulation, which may relate to either pre-morbid personality or lesion site. The paper also discusses the way in which patients of this type bear the very *status* of psychotherapeutic work with profoundly amnesic patients. Where others have seen barriers and *in principle* problems in working with such patients, we see many opportunities.

“*in a case like this. Do whatever your ingenuity and your heart suggest. There is little or no hope of any recovery in his memory. But a man does not consist of memory alone. He has feeling, will, sensibilities, moral being – matters of which neuropsychology cannot speak… Neuropsychologically, there is little or nothing you can do; but in the realm of the Individual, there may be much you can do.”*

*Aleksander Luria in a letter to Oliver Sacks*,

*from the story The Lost Mariner [* *(* *1* *)* *, p. 45]*

## Introduction

How does it feel, how does it feel? To be without a home Like a complete unknown, like a rolling stone. Like a Rolling Stone (2).

### Psychoanalysis and amnesia

The clinical phenomena surrounding profound amnesia, following acquired brain damage, provide an opportunity to gain valuable insight into the neurological basis, and the neuropsychological mechanisms, of memory and learning (3–5). Arguably, the most important finding is the existence of multiple independent memory systems [(6), p. 67, (7–12)]. Individuals who present with profound amnesia—most notably following medial temporal lobe damage—are significantly impaired on explicit recall of new episodic events (13–15). However, these people simultaneously appear to preserve their ability to retain and utilize information from other sources, such as the implicit memory systems associated with procedural and non-declarative memories (16–19), or when the information is emotionally salient (20–22). Thus, people with profound amnesia are not only able to experience emotions (23, 24), but more importantly, they demonstrate the capacity to *learn* about the emotional value of experiences, and generate decisions based on the emotional valence (22, 25–28). Importantly, this capacity is crucial to the ability to develop and sustain interpersonal relationships (29–32).

The existence of independent memory systems holds obvious importance for the theory and practice of the psychotherapies (33–38), and has been especially discussed in the psychoanalytic psychotherapy literature (39–42). For example, these neuropsychological findings align with recent psychoanalytic research and theory regarding the role of conscious and un-/non-conscious processes associated with psychic change (40, 43–45). Therefore, it is reasonable to suggest that psychoanalytic therapy with profoundly amnesic individuals can aid in the understanding of these memory systems, and their relevance for psychoanalytic theory and practice.

There have been many hundreds of reported cases of profound anterograde amnesia in the context of preserved executive functioning and working memory (46). However, very few cases have ever been reported of amnesic patients in psychotherapy [(47), c.f. (48, 49)], and only once for a long-term psychotherapeutic intervention (47). In this case, the lesion site was bilateral, involving both hippocampi and associated medial temporal lobe structures. In this first case we reported that the patient (JL) seemed to show an ability to develop a therapeutic alliance, a working relationship with the therapist, perhaps using emotion-based learning rather than episodic memory. Importantly, these findings confirmed the relevance of implicit, or perhaps more correctly, affect-based memory systems, to transferential phenomena. In doing so we extended the work of Turnbull et al. (49), who offered preliminary evidence suggesting the preservation of transference phenomena in individuals with profound amnesia.

Several papers show that a therapeutic relationship can be *present* in a clinical setting (22, 27, 49–51). However, the data reported by Moore et al. (47) were novel in that they demonstrated for the first time that transferential feelings in this population can *develop* across the therapeutic process. It also offered a description of the main features of counter-transferential phenomena when working with profoundly amnesic patients. In doing so, the paper also showed that such information can be used to adapt psychoanalytic tools and settings. This was instructive in better understanding classic psychodynamic processes, such as hate (52–59), sadism (60–64), and narcissism (65–70). The observations in the 2017 case also showed the value of employing a relational framework in patients with amnesia, particularly regarding the (often nuanced) transition between part and whole-object forms of mental functioning.

In this paper, we report a second case, in which the person was also profoundly amnesic, and had many properties in common with the case reported in Moore et al. (47). Importantly, in this new case (GA) the lesion site is different, following lesion to the anterior fornix, which was sectioned during a procedure to remove a colloid cyst. This opens the question of why these two different lesion sites should lead to the phenomenon of profound episodic memory impairment? This is presumably because of the structures identified by Papez (71) as linked: from hippocampus; to fornix; to mammillary bodies; to anterior thalamus; and to the cingulate gyrus.

Many studies have shown that amnesia is produced after bilateral lesions to *any* of these sites (72–78). However, they have also shown that there can be some variation in the nature of the amnesia and its associated presentation. One of the most notable of these differences is the presence of confabulation, which appears to be absent after the hippocampal lesions, but is often present in patients with more anterior lesions to the Papez circuit (79–83) and in particular bilateral lesions to the fornix (84–90). However, we have never been able to compare two patients with different lesion sites in a psychotherapeutic setting. We believe these two cases are unique in the history of medicine in reporting a longstanding therapeutic relationship.

## Methods

### Patient

GA was 19 when he underwent a surgical procedure for the removal of a colloid cyst. As part of a craniotomy procedure, the anterior portion of GA's fornix was sectioned. As unfortunately may happen in these cases (91–98), post-surgery GA was left with a dense anterograde amnesia, in the context of preserved working memory and higher executive cognitive functions. In addition to this, he had hyperphagia, which suggests a degree of damage to his hypothalamus, perhaps as a result of pressure from the colloid cyst (99). GA appeared to have a high degree of insight into his memory problems, his hyperphagia, and indeed his brain injury in general.

GA then moved to a residential unit. Seven years after surgery he was referred (by a neuropsychoanalytically informed clinical psychologist in his Brain injury service, who was aware of the previous 2017 case) to the first author (PM) for psychoanalytic psychotherapy. It was felt that GA might benefit from an opportunity to explore and process the emotional consequences of the devastating and life-changing consequences of his brain injury.

### Brain injury service report

A document, compiled by GA's Brain injury service immediately before the referral, reported that GA had a tendency toward irritability and aggressiveness, and he could spontaneously become tearful and depressed. The report also noted several elements of GA's presentation before psychotherapy, which are dramatically different from his presentation in the psychotherapeutic setting (see below). The first is that GA's mother was terminally ill at the time he was diagnosed with the colloid cyst, and that she passed away shortly after his surgery and subsequent brain injury. The report noted that GA attended his mother's funeral, but has no memory of this, nor is (*sic*, see below) he aware of his mother's death. The second issue in the Report is that when GA is asked how old he is, he invariably replies 18 or 19, again a finding of note in relation to his presentation in therapy.

A final matter of interest in the Report is the highlighting of GA's hyperphagia: his persistent request for food, requests that are so persistent that they feature as a top priority in GA's Individualized Educational Aims (a treatment plan) and behavior management schedules. Staff were advised to refer GA to a mnemonic device that reminds him of the day's schedule and mealtimes, and to orient GA to the time of day relative to the schedule. The report also concluded that there were no pre-morbid psychiatric issues present in GA's medical history.

### Psychoanalytic psychotherapy

GA's treatment consisted of 56 once-weekly psychoanalytic psychotherapy sessions, across a period of 18 months. The sessions took place at the same time and day of the week, every week, at 11 a.m. on Friday mornings, with the exception of typical holiday breaks and occasional missed sessions owing to illness or the like.

In the first session, following the initial consultations GA, was invited to use the couch. GA had commented upon, and appeared to be curious about, the couch in the initial consultations. This is an issue discussed below in relation to his amnesia. GA accepted the invitation. The sessions were audio recorded and transcribed.

## Results

As one might expect, GA's case, and its treatment, bears on a number of complex issues. We have tried to summarize these around six broad themes.

### Differences and similarities to the previous case

As discussed above, GA's post-surgical damage, primarily in the fornix, represents a lesion site to a quite different part of the Papez circuit to that of JL (47), whose amnesia followed from damage to hippocampi and surrounding medial temporal cortex. Nevertheless, because of the central role of the Papez circuit in recent episodic memory, GA's neuropsychological presentation was similar to that of JL's. Both patients had a profound anterograde amnesia, in the context of broadly preserved working memory and executive function. However, there were some differences, and it is interesting to explore (see section Discussion) whether these are due to the different lesion site, the severity of the amnesia, or differences in premorbid personality.

One area of difference was that, for GA, there appeared to be less frequent periods of silence in sessions. Where they did occur, they were also more brief in duration to those of JL. GA also appeared to be more demonstrative emotionally than JL, for both positive and negative emotional experiences. GA could be ebullient and animated when he was happy, or excited when he was speaking about something holding a positive affective charge for him. During periods of negative emotional affect, GA appeared to experience much deeper regressions to more primitive affective states than JL. For example, on several occasions, while talking about his mother's death, GA reported how thinking about this made him feel sad. Soon after he would be gripped by the most profound sadness. It was clear he was deeply distressed at the thought of losing his mother.

The painful expression of his grief was so raw and unprocessed it was almost as if it were his first time to hear the news of her death, which of course in a sense it (repeatedly) was. Conversely, JL who most certainly had much to be sad about [see (47)], given bereavements and family separations experienced by him, he did not display anywhere near the same level of affective response in relation to talking about them. If anything, one might say he was strongly defended against the difficult feelings, and when probed, he would play down the impact of these difficult emotional experiences upon him.

A second difference was that, for GA, there seemed to be a pre-dominance of primary process thinking [(100), pp. 339–341], in contrast to JL, who might be said to have more capacity for secondary process [(100), pp. 339–341]. Notably, GA used scripts, or what seemed like rehearsed narratives, apparently in a symbolic way. These were old repetitive scenes, with minor alterations (which may have been indicative a minor level of confabulation, see below). For example, the following was a frequently used script:

Session 1, 5 min in—

GA: I would have been going to school with some kids from like the absolute wealthiest families in the world. There was this Muslim lad LG… his dad was both an oil sheik and a diamond miner. They wouldn't have been billionaires, they would have been trillionaires. LG would have been lavished in extremely expensive gifts and presents. For his seventeenth birthday he got a range rover jeep and I think he only had that for only four or five months and then he traded up for a, I think it was a two seater Ferrari or an Astin Martin.

Later on in the same session (approx. 10 min)

GA: … I remember when I was going to school up here in _____ at the ____ like there was this Muslim lad called LG he got a Hummer jeep I think it was like for either his seventeenth or his eighteenth birthday like his dad had the most wealthiest job in the world he was both an oil sheik and a diamond miner.

And again further along in the same session—

GA: …. Like I remember there was this one Muslim lad in my year up here in _____ at the ______ like he would have came from a serious, serious, money family… like his dad was both an oil sheik and diamond miner like they wouldn't have been billionaires they would have been trillionaires… like for his seventeenth birthday what do you think daddy got him?

P: I have no idea.

GA: A Ferrari 911… How long do you think he had it before it was nicked on him?

The themes of these repetitions could be regarded as belonging to two major categories, of positive and negative.

Sometimes, GA *did* display the capacity for secondary process thinking. This typically happened when the therapist was able to retrieve his *own* capacity for thinking from the (often mind-numbing) effects of listening to the scripts repeatedly, sometimes upward of five or six times in a row. The therapist might have an original thought in relation to the material, which then had the effect of breaking the momentum, and altering the cyclical nature of the scripts. One such script which was often used by GA related to what he was going to do on the weekend. His session was at 11 a.m. on Friday, after which he usually returned to his family home for Friday evening and Saturday morning, after which he returned to the residential unit on Saturday afternoon.

GA had what was most likely a rehearsed set of semantic facts about what usually happens on the visit home, and this script was often used to restart conversations after a period of silence. Again, practically the same content with slight variation, and usually positively valenced affectively. After one such deployment of the script GA's therapist had the sudden thought to enquire as to whether there was anything he did not like about the visit home and the following interaction ensued:

PM: Is there anything that you are not happy about the weekends?

GA: No not really… I'd like to have longer time at home I'd love if it was Friday night and Saturday and come back on Sunday afternoon after lunch at home.

PM: Have you ever asked about that?

GA: I have, it's not possible.

PM: Why have they said it's not possible? Who has said it's not possible?

GA: My dad.

PM: Your dad?

GA: Yeah.

PM: Ok. Well what reason does he have for that?

GA: Like the schedule that they do in (residential unit) like it only works that I only have one night at home at the weekends if I was to have Friday and Saturday it would break up the schedule at_____ there would have to be a lot more work done with me, rehab work back in ______ to keep me on the same track as just having one night at home. It's fine I take that on board, like I would I would like to be at home full time as opposed to just Friday night at home and back at Saturday after the lunch.

PM: Have you asked them about that?

GA: I have it's not possible. I've asked both my dad and xxx staff.

PM: Ok.

GA: It's just not possible.

PM: That's really disappointing.

GA: Ah it is indeed but what can you do? If I was to get angry and upset about it it would just add to the problem and an answer to that might be not coming home at all and I wouldn't be able for that.

PM: You feel that you might be punished if you were to ask? And not allowed home?...If you were to be angry or upset with them about it?

GA: Yeah like say if I was on to them to stay at home on Friday night and Saturday night and like if I was to be quite repetitive they would be like look we've been through this we've told you this it's just one night at home is what you get and you know the reasons for this.

PM: So this has happened has it?

GA: I think so Paul.

PM: So you have asked and you have become upset about it?

GA: yeah.

PM: and was it said to you that you wouldn't have any nights at home if you didn't stop?

GA: no that hasn't happened.

PM: But you're fearful that that might happen if you were to express… (GA and therapist talk over each other here)

GA: Yes

PM: …you know you're being upset at wanting…

GA: …wanting to be at home for longer, yeah.

PM: Mmm.. but that's very difficult G.

GA: Well it's fine Paul like I've been in ____ (residential unit) for years now.

PM: But is it really fine? You know? Because it sounds to me like it is something that you get upset about?

GA: It is Paul.

PM: It is something you get angry about and you feel like you're not allowed to express that … and there may be a punishment if you were to do that?

GA: Sorry (begins to sob).

PM: It's really upsetting.

GA: It is Paul sorry.. (uncontrollable wailing).

PM: It must be so difficult.

GA: (continues to wail) sorry.

PM: It's ok … it's what this space is for. (GA begins to blow nose vigorously)

PM: it must be so difficult GA.

GA: It is Paul it always seems to go so fast when I'm at home with my dad.

PM: It must be so difficult to have to turn around and go back so quickly?

GA: It is but we pack an awful lot in to our time at home. Like after our session here … it's ten to twelve we finish today Paul isn't it?

PM: Yes.

The sudden thought arising in the therapist's mind, when articulated, seemed to bring GA and the therapist into a new space therapeutically, a space where the therapist had the feeling that interaction was more reciprocal, and both he and GA were in the process of meaning-making.

This stands in contrast to JL, the previous case. There, these spaces were more present, and accessed more easily [(6–12, 14, 47), pp. 3]. JL did not seem to use, or *need* to use, the mechanism of the rehearsed scripts. Instead, JL could access this state of mind more easily, though he did struggle to hold on to it. In contrast, GA seemed to struggle to access secondary process thinking, though *could* manage this with the therapist's help, and then use it quite well.

GA: No it's ok… I remember when I was going up to school in ______ up at the ______ there would have been kids in my year say their dads would leave the house at half five, six in the morning and they wouldn't be back until say half ten or eleven at night and they do that at a minimum of five days a week sometimes six.

PM: So they wouldn't see their dads?

GA: No they wouldn't at all.

PM: A bit like how things are for you now.. you don't see him every day but you see him on the weekends.

GA: Yeah that's it.

PM: What is that like for you?

GA: Ah no it's fine I'm I'm, I'm, hap.. hap.. happy with the way things are. We always have absolutely fantastic weekends. Like I'll be staying at my home house tonight and I'll have my dinner at home like we'll probably do an activity tonight like go out to a film or go bowling or visit one of my dad's friends or something like that and then tomorrow morning we'll go for a swim in______ (local swimming pool) and we'll do sixteen or twenty lengths then I'll have lunch at home and then head back to _______ (residential setting) then after lunch.

PM: You miss your dad.

GA: I do indeed.

PM: Sounds like you're very close.

GA: Well we've been very close I'm close with both my parents with both my mam and my dad but my mam is gone now.

PM: That's right yes and what's that like for you?

GA: It's very very hard like.

PM: It must be.

GA: Like because like say I know my mam is gone but I don't know the date like today was it 2010 that she died?

PM: I don't know that G.

GA: It was around then I think.

PM: Was that before you had the operation or after?

GA: After.

PM: Ok.

GA: No it was either 2008 or 2010 when I had the operation sorry I don't know (becomes visibly upset)…. is the weather due to be like this for the weekend or do you know?

PM: It's supposed to be good it's given good.

GA: Up until when?

PM: Just the next few days is all I heard.

GA: Friday Saturday Sunday?

PM: I'm not sure I think so.

GA: Sorry.. it would be great if it was wouldn't it?

PM: Mmm.. you were starting to get into telling me about your mam and you got quite upset … it's as if you moved away from it?

GA: Sorry … I didn't mean to.

PM: I'm not being critical.

GA: I know … being honest with you like …say each morning when I wake up I wouldn't think that my mam is gone. Say if I was waking up at home it is nearly in me to, say, go up to her and say will I put the kettle on for a cup of tea or are you ready to make a start on the breakfast or something like that…

PM: And then you realize …

GA: That she's gone…

PM: That must be very difficult.

GA: It is but it's happened for so long now that it's…I'm kind of use to it in one way.

PM: It's still like a shock when you…

GA: Yeah like nobody has ever spoken badly of my mam or spoken ill of my mam like if anyone is speaking of my mam they're speaking words of praise like you know.

PM: Mmmm

GA: Like she helped me out with this she helped me out with that. She did this for us she did that for us. Like that was just the kind of woman my mam was she would go out of her way to help out other people like and to make other people succeed in all different things.

These issues are addressed in more detail in the Discussion section, and may relate to differences in the premorbid ability between the two patients.

### Counter-transference phenomena

In the area of counter-transference the therapist could at times experience an overwhelming range of oscillating emotions. Oscillations between the poles of pronounced feelings of powerlessness and helplessness to feelings of exhilaration, aliveness, and caring toward GA. This fluctuation of emotional states could make it difficult to “stay” with GA at times. The countertransference phenomena experienced by the therapist fall into three broad categories (a more extensive and detailed selection reproduced from the therapist's notes can be accessed in Appendix C, as we feel this is important technical information which will be of value to therapists in their clinical work with this population).

The first was a sense of confusion. For example, the therapist noted: “*I find myself spending a lot of time wondering about how to respond to the repetitive (scripts) questions I have heard many times before.”* These were questions the therapist had heard in all of the previous sessions, and could take up at least half of the session. Over time, the therapist realized the importance of holding these questions in mind, and in particular noticing the level of urgency with which they were asked, to better establish the emotional tone. In a typical session, one or two pieces of information of this type might come available to aid understanding.

One such example is a session where the questioning was especially urgent in tone and occurred at a very high frequency. GA had been accompanied to the session this morning by a new healthcare assistant who was unfamiliar with the protocols of timekeeping in the therapy, and there was a strained interaction between the therapist and the assistant over the time when GA should be collected. The therapist was left with an uncomfortable feeling in relation to the healthcare assistant, and in particular their suitability for this type of work, and for the subsequent effects for GA. Reflecting upon this context was helpful to the therapist, and seemed to provide an explanation for the added intensity and urgency in GA's repeated questioning. Across time, the therapist learned to trust this phenomenon: that it was not so much the explicit content of the question that was important, but the emotional package that wrapped around it. Attending to this, and opening up enquiry about it, also seemed to be helpful for GA.

A second phenomenon was a sense of frustration or irritation. The therapist noted: “*When he asks me if I think something is ‘too much' he can be insistent and repetitive and this evokes an annoyance/frustration in me.”* This typically occurred after one of the rehearsed scripts had been deployed, which was usually upwards of six or seven times in a session. The repetitive nature of these scripts had a mind-numbing effect on the therapist, who could very often find himself in a kind of stupor. GA's insistent asking if something was “too much” seemed to be a means through which he might “wake” the therapist up. The therapist eventually came to understand this as GA's way of regulating a powerful anxiety, related to the uncertainty lying behind his rehearsed scripts. It also had the effect of kick-starting the therapist's ability to again think originally (or without stupor), and brought the therapeutic work closer to the realm of secondary process thinking.

The final transference phenomenon was a sense of care, and “parental” attachment. The therapist noted*: “When GA says he likes Fridays because he gets to come here and talk to me I feel very close to him.”* On another occasion: “*The thought that he may not at times be aware that his mam has passed away can feel terrible for me to know this – like having to break the news to him for the first time.”* These feelings were much less frequent than the first two types, and also developed gradually across the therapeutic process. Notably, the parental countertransference would typically follow a period of secondary process interaction and meaning-making, where some affect had been brought in contact with a remembered event in GA's life. An example of this occurred at the end of most sessions where, unusually for the therapist, he would find himself “fussing” over GA: reminding him to put his coat on, or checking that he had all his belongings before leaving.

In contrast to these three types of common countertransference phenomena, there was one category in particular that was *absent*: the so-called “organic” amnesic countertransference phenomena that we have reported elsewhere (101, 102), such as increased forgetting by the therapist. These did not seem to be a prominent feature in the work with GA, where they had been a common occurrence in the work with JL [see (47), p. 10]. In the case of that work with JL there were many instances of the therapist, and the broader circle of professionals involved in his care, unexplainably forgetting, and struggling with their otherwise intact memory. JL's therapist also experienced a high incidence of lethologica (tip of the tongue forgetting) in JL's presence, for topics he otherwise would usually have reasonably good access to. Organic amnesic transference also occurred in the brain injury service's dealing with JL and in the residential setting: staff working with JL also forgot about him in many different ways, and with an above average level of frequency. However, these phenomena were absent with GA.

### Hyperphagia

A cardinal feature of the brain injury service report was the hyperphagia that GA experienced. Indeed, to the extent that it formed the focus of his cognitive rehabilitation programme. The residential unit staff noted that GA asked with excessive frequency, the questions “When is mealtime?,” “Have I had breakfast? I'm hungry,” “When are we having something to eat?.”

Amazingly, upon review of the audio recordings of 56 sessions across 18 months, there is not a single instance of GA asking about mealtimes, or saying that he was hungry. The therapist was not aware of this aspect to GA's presentation at the outset of the treatment, and only became aware of it as a result of a communication, updating all staff involved in his care about a new individualized education plan to address his excessive checking about mealtimes. It presented an interesting technical challenge to the therapist as to how to manage such information from the patient's life outside the therapy sessions. This is explored further in the Discussion section.

### Superficial awareness vs. lived experience

At times GA displayed quite a remarkable ability to retain information relating to historical events. However, it would soon transpire that on the surface, while *appearing* as having an awareness of the event, the awareness was superficial, and GA did *not* appear to have the ability to use or integrate this information in to a lived experience in the here-and-now (103), at least where the historical information was relevant to the current situation. One such instance of this recurred around the theme of his mother's death.

GA: well every now and then I can get sad – I miss home and I miss my mam – It's hard being in ______ all the time. I only get to go home for a small bit at the weekend. Don't get me wrong it's great but it's not long enough, I'd like to stay at home for longer – it's hard because when I go in the front door I am looking forward to meeting mam – and then my dad tells me that she has died.

PM: that must be so terribly hard for you.

GA: it is.. (GA breaks down there is a deep booming, harrowing, wail. It is raw unprocessed affect which after a short period of time, approximately 1 minute, dissipates as quickly as it erupted).

This was not an isolated incident, as the reader will see later in the Results and Discussion section, there were many such instances. It highlights the importance of being able to experientially (or episodically) work through a traumatic experience such as a bereavement. The working through/mourning process provides a level of protection to the person against the affective intensity of this terrible new reality involving the loss of a loved one. However, the superficial understanding possessed by GA is apparently not strong enough to protect him from the affective pain, which so easily erupts in him on encountering reminders of his mother's death.

### Procedural abilities

Despite GA's profoundly impaired memory, he demonstrated the capacity to acquire new *procedural* abilities. For example, upon entering the room he almost invariably left his bag and jacket on an armchair, and next went to lie down on the analytic couch. GA had been informed of the option to use the couch at the initial consultation stage of the therapy, and then invited to choose the chair or the couch at the beginning of the first psychotherapy session. He chose to use the couch, and continued to do so for the remainder of the treatment—with one notable exception, where he appeared to be unusually distressed.

During this session where he opted to use the chair instead of the couch he did not comment upon it, and took his seat in the armchair facing the therapist. It was notable that he was upset and distressed that day. During a stressful episode later in that session, GA asked whether he could go to the toilet. This was reminiscent of the psychoanalytic literature on evacuation (103). GA asked to go to the toilet during many of the subsequent sessions, sometimes twice, and often following a particularly after emotionally difficult subject matter. Interestingly, this was also an incident of “one trial” learning in the domain of procedural memory, as GA remembered his way to the toilet.

At the initial stages of the therapy, GA was met at the door of the building, and guided to the therapy room by the therapist. Similarly, when the session had ended, the therapist escorted him out to the front door of the building. After several weeks, GA had acquired the procedural ability to navigate these journeys without the assistance of the therapist. Interestingly, given his profound episodic amnesia, GA also remembered the therapist's name in a matter of weeks, and used it unprompted for the remainder of the treatment. There were also facts about the therapist's life that GA remembered. He remembered the fact that the therapist had played rugby and, similarly to JL, that they both had an interest in sport. GA also remembered the region and county that the therapist resided in, and would refer to it appropriately in conversation.

The foregoing are examples of newly acquired procedural abilities. However, GA also demonstrated what we presume to be limited *episodic* memory alongside procedural memory: a capacity for remembering some names and facts about other residents he shared the residential unit with, which seemed to be consistent and sustained over time. This episodic memory ability was very limited, as GA did not recount *any* episodes of shared experiences or interactions with his fellow residents.

### Repetition

Repetition is a frequently observed phenomenon in psychoanalytic therapies (104). These repetitions can manifest in *inter*personal and *intra*personal patterns of behavior, known in psychoanalytic parlance as transference and countertransference relational dynamics (105).

In the following section a sample of clinical vignettes, derived from psychoanalytic psychotherapy sessions with GA, will be used to illustrate the three distinct forms of repetition, demonstrating further the unique phenomenology and function of each class of repetition (The reader is referred to Appendix B, where more comprehensive versions of the vignettes may be accessed). For further information relating to the differing types of repetition, the reader is referred to Moore et al. (47), and to Appendix A of this paper where an abridged description is available.

#### Regulatory (fixed) repetition

As with JL (47), there could be silences and pauses in the therapy process. It was, like JL, difficult to know at times if these breaks in the temporal continuity of the sessions were organic (i.e., a result of GA's brain injury and memory impairment), or defensive. Defensive, in the sense that the therapy process may have become emotionally overwhelming for GA, so that the topic was dropped and a silence ensued. GA also used a similar mechanism to JL in an attempt to restart the therapy process. This mechanism we have identified as a *fixed* form of repetition, where GA used a question relating to a familiar theme as a “springboard” of sorts to resume the interaction with his therapist. Some brief examples are provided below:

Session 3

after a brief pause—GA: it's a beautiful day out there now Paul.

Session 4

1 minute pause—GA: so, what's the hottest place you've ever been?

Session 24

brief pause—GA: do you know what the weather is going to be like over the weekend?

Session 25

Upon entering the consulting room—GA: it's a beautiful day out there.

Later in the same session after a difficult discussion relating to his dad, and a brief pause—G: do you know what the weather is supposed to be like for the weekend Paul?

Session 48

Again upon entering—GA: it's a beautiful day Paul …. Is it to be good for the weekend?

The issue of Fixed Repetition is readily illustrated by the repeated use of questions about the weather. Indeed the therapist came to think of the weather as a proxy for GA's emotional status. When GA said that the weather was good, it might mean that he was feeling good, and *vice versa*. In exploring with GA the varying extremes of weather, the therapist wondered whether that might actually be an exploration of what range of emotional experience he (the therapist) might be able to withstand? And when GA asked him “Do you know what the weather is to be like for the weekend Paul?”—He might really mean “What mood is my Dad going to be in? What will the emotional atmosphere be like at home?” Would GA be entering an emotional maelstrom, or will the home climate be calm?

As can be seen the first category of repetition regulatory or Fixed Repetition, was characteristically unchanging in nature, with no variation of structure or content in the clinical setting. This class frequently appeared after the disruption of temporal continuity of the sessions, the onset of which was indicated by the dropping of content, or cutting off of conversation topics, often mid-flow. It was not clear at times whether this was due to the “organic” brain injury, and the episodic memory system resetting itself due to some new stimulus entering consciousness and dislodging the current train of thought, or if it was due to a defensive operation to protect GA from becoming overwhelmed.

#### Knowledge (epistemological) repetition

The repetition of explicit content which was not rigid but variable, and appeared to revolve around a fixed context, usually an event experienced by GA outside of the therapy session. These external situations puzzling and confusing to GA. These contents were (amazingly, given the diagnosis of profound amnesia) brought repeatedly to the therapy sessions. Broadly speaking, it seems the function of the revisiting of these experience was to make sense out of the confusion. We believe the repeating and revisiting of these topics across the treatment period is a function of the developing therapeutic alliance, and the transference–countertransference relationship between patient and therapist. We have summarized a select sample of this type of repetition below for the reader. The unabridged vignettes can be viewed in Appendix B, and the reader is referred to them for greater context and detail about this clinically fascinating phenomena.

Week 8 of therapy and subsequent to a discussion about psychotherapy:

After introducing the broad topic of mental health and how classmates in college attended different mental health professionals, the therapist enquired as to what it was like for GA to be in therapy. GA replied that it was ok, and that he was good enough most of the time, except for some ups and downs. The therapist invited him to say some more about the ups and downs, to which GA introduced the topic of going home on the weekends and how he missed home and missed his mother. He went on to say that he looked forward to the weekends, and returning home to see his mother, only to be met at the front door with the news from his father that his mother has died.

There were many such incidents throughout the course of the therapy, where GA introduced, unprompted, the fact that while he knew *semantically* that his mother was dead, he nonetheless had this dreadful experience of experience of looking forward to seeing her on the weekend, only to be informed of the fact that she was dead. However, across the duration of the treatment, there did seem to be a lessening of the intensity of affect experienced by GA in connection with the retelling of the experience. For example, in week 26 the topic is revisited, after GA spontaneously tells the therapist that he “likes coming here” and “…I love the couch, and I get to talk about whatever is troubling me.” When the therapist asks if there is anything troubling GA at that moment, he responded that he has been thinking about his mam a lot and really misses her, and then begins to cry uncontrollably. The therapist notes that during this episode GA's crying does not seem to have the same level of intensity affectively speaking as previous times, or indeed the first time the topic was introduced.

There is a marked difference in GA's affective response to the issue of his mother's death at week 48 toward the end of the treatment. During the session he comments that he wanted to work hard to improve his memory to resume his university studies, as his mother would have wanted that. GA did get upset as he mentioned his mother and told the therapist that he missed her. However, it was not the loud booming cry that was present at the beginning and earlier in the treatment it was much more subdued. When the therapist enquired as to how he now felt about his mother's death he replied “Ah it's still hard, you know, but it's getting easier.. I still miss her when I go home, but I don't expect to see her as much… and that's a lot easier for my dad.”

Thus, remarkably, there appears to be learning about this critical event at some (semantic and procedural?) level, but GA is still caught off guard on an episodic or autonoetic level, especially as regards the link to feelings. It is as if GA can think about himself in the third person, but not in the first. He can remember things that happen to other people, and to himself only as facts or information told to him *by* other people, but does not seem to have the experience of being there.

Interestingly, this phenomenon in some way shines a light on GA's use of the repetitive rehearsed scripts. How might this be understood? Firstly, GA is impaired in his ability to lay down and retrieve novel episodes, and he therefore repeats scripts. During some of the repetitive scripts a thought would enter the therapist's mind, and stir him from the often paralyzing boredom of the repetitions. This appeared to provide a link of sorts, and the therapist would then enquire about it with GA. This had the effect of slowing GA down, and allowed him exit the repetitive loop of the script. Helpfully, the introduction of the link by the therapist usually led to a productive interaction between GA and his therapist. Similar to the work with JL (47) this shift from primary to secondary process thinking was underscored by the developing positive transference relationship, without which it was unlikely the rigidity would have subsided. However, it appears that the affective link needed to take place first, better developing the relationship.

#### Implicit repetition

Implicit repetition refers to the repeating of unconscious transference themes, possessing a clear relevance to current events in GA's “here and now” life outside of the therapy session. These are themes which were not expressed explicitly in the therapeutic process, but none the less communicated implicitly in the subtext of the explicit narrative with the analyst. This classically psychoanalytic approach to attending to the patient's narrative and session content (106–115) facilitated GA in transforming primary process material to a secondary process level of mentation.

One such example of implicit repetition in the clinical work with GA is provide here below:

GA: When I was in College one of the kids whose name was LG came from an extremely wealthy family, one of the wealthiest families in the world – his dad wasn't just property tycoon, he was a really wealthy stockbroker, and a diamond miner. He wasn't a millionaire, he wasn't a billionaire, he was a quadrillionaire. My mate didn't get a hummer jeep for his eighteenth birthday, he didn't get an Audi jeep, a BMW 5, he got a Ferrari – that's too much isn't it Paul? It's excessive isn't it Paul? too much for a young fella like that. If god came down in the morning and said G you can have all that I'd say no you're grand, you're alright thanks, I'm happy with what I've got thanks.

PM: It does sound excessive G and might be more trouble than it's worth.

GA: I wouldn't like it to be honest Paul, to be that rich, the responsibility would be too much. You couldn't enjoy your life with that much money. You wouldn't know if people were just hanging around with you for the money or because they like you.

PM: I'm thinking about how you might know how he feels on some level – might it be like that sometimes for you in _____ (residential unit)? You know are people really friends with you or are they just looking after you for the money?

GA: Well I like _____ (residential unit), but it's no place for a young lad like me. The staff are nice but they are only moving through the place and they could be there one day and gone the next and there is a completely new person there to get to know. I have some great friends at home (continues to tell me all about his friends and how they all go out together).

However, GA's father has told the therapist that his friends have stopped calling for him when he is home, and have also stopped visiting him in the residential unit for nearly 4 years now. It is also a reflection of GA's mind, and reflects an unconscious awareness of what has been lost. GA sees all these brilliant “Ferrari” minds around him, and rationalizes that he's probably better off not having one of them, it would be too much trouble anyway! The rich students live in a completely different world to him, and he attends school with them every day. This may also be a reflection of the difference between his mind and the therapist's mind, and the minds of people he comes into contact with everyday who care for him. Indeed, GA might also have been wondering whether the relationship with the therapist was authentic, or was it just because he was getting paid to do this job.

Identifying the implicit themes contained within GA's explicit narrative also allowed the therapist to open up, and explore, topics that otherwise might not have entered the domain of the therapy. For example the therapist was able to explore with GA the nature of their relationship in greater detail as a result. Thus, providing an opportunity to deepen and strengthen the therapeutic relationship.

## Discussion

A case as complicated as GA's raises a number of issues. These are discussed below in three main categories: Phenomena specific to GA and JL; Phenomena unique to GA; and Recommendations for technique.

### Phenomena specific to GA and JL

The most important issue is forgetfulness. Both GA and JL had a level of memory impairment, as a result of their brain injuries, that was evaluated by neuropsychologists, and experienced by the therapist, as profoundly amnesic. In reality, this meant that, unprompted, neither were able to retain or retrieve memory after 30 s or so. Both could sustain a conversation. However, as soon as the temporal continuity was interrupted, they were unable to pick up the conversation from where they left off. It was notable in both that sometimes the disruption in temporal continuity could happen “organically,” as a result of the amnesia. At times a disruption appeared, often when the emotional content of the conversation became overwhelming, where we might regard the process as one of defense? Interestingly, in both cases the (unconscious) strategy of Regulatory Repetition (discussed further below) was employed, to reinstate connection and continue conversation with the therapist.

Notably, the ongoing disruption to the temporal continuity of the sessions also made it *very* difficult for the therapist to maintain focus. In both cases, the attempts to reinstate continuity, and in particular their repetitive nature, had the effect of inducing a type of “mind-numbing stupor” in the therapist [see (47, 116)]. When the therapist was able to eventually emerge from the trance-like state, a more immediate and present connection could be achieved, where some therapeutic gain might be possible.

What seemed to produce the therapeutic gain, and build the therapeutic alliance, in both cases, was a personal quality of “affability” present in both GA and JL. They were both very friendly and likable people, who seemed to enjoy the company of others. As the relationship developed, they frequently liked to joke with, and tease, the therapist, in a playful way (described in Ireland as the “*craic*”). At times this could feel quite child-like. This child-like affability gives rise to the question as to whether there might be a defensive function to the quality of the interaction? If GA or JL's temporal continuity was so disrupted, it seems worthwhile to speculate whether, subjectively, there was a certain vulnerability in having to constantly reorientate oneself in time and space, every 30 s, or when the continuity of an interaction was lost. In other words, both patients might need to keep the people they are interacting with “good” [c.f. (117)].

Interestingly, this affable quality might be a major factor, for both, in contributing to the ability to develop a transference relationship with the therapist. Quite early on in both cases, each person was able to explore issues that were puzzling to them. These were issues that necessitated them to be in a vulnerable position in relation to the therapist, and subsequently allowed them to “work through” to a better understanding. In psychoanalytic parlance, this refers to the whole-object representation of the issue (118). It seems plausible that this capacity to be vulnerable and open to developing a new understanding of an issue, might be a function of the ongoing developing transference relationship between patient and therapist.

Notably, similar types of repetition to those identified in the treatment with JL were also found to be present in the treatment with GA. We have termed these Regulatory, Epistemological, and Implicit repetition, and were all evident in the therapeutic work, in both cases. These phenomena might be thought of as iterations of primary process mental functioning. This process of perpetual disruption limits the ability of executive function to generate a reliable version of reality. Thus, we are permitted an opportunity to glimpse what might be considered to be the unconscious motivational circuitry in action.

These repetitions might also be regarded as manifestations of the SEEKING system (119). It is interesting to reflect upon why a person needs to repeat anything at all, and in doing so arrive at an old psychoanalytic idea that the objects which satisfy our biological needs are situated outside in the external world (106). Survival is contingent upon being able to connect and engage with the external world, and the SEEKING system, always active, provides the maximal opportunities to meet the object and therefore ultimately the need. Later in the Discussion we cover these repetitive phenomena as they appeared in GA's treatment. In the Recommendations section we address implications for technique in relation to the persistence of these repetitions in the service of the SEEKING system in persons with a brain injury, and profound amnesia.

Both GA and JL employed the protective strategy of a narcissistic defense (65) in order to maintain a sense of order and control in the context of, what we can only imagine, must be the terrifying and disorienting chaos of the profound amnesia they both experienced. When the topic of the therapy moved in the direction of an issue that was emotionally difficult, there was often a subsequent closing down, a not taking in, or even a pushing back, against a newly suggested insight or perspective. Understandably, this too was a function of the developing transference relationship between patient and therapist. In a range of areas, across a variety of issues, both GA and JL can be seen to follow the *same* relational dynamic pattern. Approaching issues in therapy circumspectly and tentatively at first, in a cautious and protective manner, which subsequently softened across time, into a more open and receptive form of interaction. This is reminiscent of recent research in the literature relating to the relationship between emotional tone and episodic memory consolidation and reconsolidation (20, 120–122).

It is also interesting to note how recent conceptual developments in neuropsychoanalysis might be applied to, and evidenced, in both cases. In particular the concept of Free Energy [(123–125)—for more information on this topic the reader is referred to Solms and Friston (126) and Solms (127)]. Essentially, as a self-organizing system, our mind-brains are motivated to minimize uncertainty and promote certainty. In psychotherapeutic work, painful affect is considered to be a manifestation of uncertainty and excessive Free Energy. With the help of the therapist, and again as a function of the developing transference relationship, GA and JL were able to bind Free Energy associated with the uncertainty surrounding an issue in their lives, leading to greater certainty/understanding and a reduction in negative affect/uncertainty. In both cases, a reduction in prediction error was achieved, with greater precision in predictions in relation to their relative issues. A more accurate representation of reality appeared to benefit both patients.

### Phenomena unique to GA

When considering clinical phenomena distinct to working with JL, and unique to GA, it is difficult to tell whether these differences are due to variations in premorbid personality, or perhaps differences in lesion site within the Papez circuit (as discussed in the Introduction). However, some striking differences are of note.

GA was more verbose than JL. JL did not seem to have the same pressure, or urgency, to speak as GA. Compared to JL, he did not seem to be comfortable with long silences, and in a way worked hard to avoid them. JL could comfortably sit in silence for up to 2 min, sometimes longer, many times during a session. In comparison, with GA's silences, it might be more apt to call them pauses, rarely lasted longer than 30 s, after which time he would reconnect, usually with an enquiry about the weather. GA's topics of conversation also seemed to be more prepared/ rehearsed than those of JL, and they were delivered in a rapid quick-fire manner, slipping off the tongue like a memorized piece of script.

While GA seemed more verbose than JL, it did not necessarily follow that he had greater access to secondary process mental functioning. Often, the rehearsed scripts seemed like ready-made packages, inserted into conversation to fill space. These scripts were also accompanied by a deadening empty countertransference experience in the therapist. In this way, we can also think of GA's presentation being more primary rather than secondary process (128–131).

Secondary process mental functioning was only achieved when the therapist could recover his own capacity to think. Perhaps to notice something “new” or different in the content, and then offer this is a possible link for GA to consider. This frequently shifted GA and his therapist into a more immediate, and what felt like more intimate, relational space—one where there was a greater possibility of making meaning. We may also view this as GA's attempt at emotion regulation, where he both expressed and experienced emotional states through the vehicle of the rehearsed script.

While it is not possible to say that this is something *entirely* unique to GA, he did, and could, use the psychoanalytic couch. Alas, as a comparator, JL did not have the option of using the couch in the location where his therapy sessions occurred. GA was informed of the option of using the couch at the outset of therapy, and chose to use the couch in every session but one (the notable exception of a session discussed in Results). GA frequently commented upon how comfortable the couch was, and how he enjoyed the experience of “talking about what troubled him” while lying down on it.

It is also interesting to consider the issue of affect, in particular how it was expressed by GA, and subsequently experienced *by* the therapist in the sessions. GA could quickly become emotional during therapy sessions: extremely sad or extremely happy. There was a fluidity and openness to these emotional expressions. With happiness, it could develop easily into an infectious and full hearty laugh. Conversely, when sad, GA could experience what seemed like a deep and painful unmitigated grief, which could last for several minutes. It was as if he was in the grip of his emotions: as if GA did not *have* emotions, but that they had *him*. Compared to JL, whose emotional world seemed less fluid and open, GA experienced and expressed affect at what might be considered an intense level. It is not clear what might explain these differences in the presentation of GA's affect. However, it is interesting to speculate whether it is related to the lesion site. GA's site of injury in the Papez circuit is a far more anterior lesion site than that of JL. How this stands in relation to damage to impulse control circuitry, and the down regulation of affective responses, remains an open question. The difference may, of course, also relate to premorbid personality, and this issue will presumably be resolved in future research.

The different lesion sites between GA and JL might also help in understanding the issue of confabulation in relation to the two cases. While both had a relatively low incidence of confabulation, compared to patients with classic Korsakoff's syndrome (48, 132–137), there was a discernable difference between GA and JL. At first sight JL, did not appear to confabulate. However, on closer inspection of the transcripts, minor confabulations, or inaccuracies, could be identified. Small details such as the interchanging of names, place, and dates, in repeated topics, across transcripts, were evident *across* sessions. In contrast, GA could confabulate multiple times, in relation to the same topic, even *within* a session.

In both cases the confabulation might be considered mild. In relation to the classic confabulation of patients reported in the literature (132–134) there appears to be the common occurrence of a “search and retrieve” error at the source of the confabulatory experiences. GA and JL's confabulation is less discernable, as it mainly involves the substituting of names, places, and times. In classical Korsakoff's amnesia, it prototypically involves the substituting of *entire* memories, erroneously for the lived experience. In these cases, it appears that the stimulus evokes a search for meaning, but due to a failure of executive function, the correct memory cannot be sourced, and an approximation is used instead.

It is interesting to consider these types of confabulations alongside, and in relation to, each other. JL's confabulation appears to be the least extreme, after a lesion site is confined to the posterior aspects of the Papez circuit. In contrast, GA experiences a more intense, but still relatively mild form of confabulation, after a more anterior lesion. Patients with Korsakoff's syndrome experience more extreme levels of confabulation, after more anterior lesions than GA and JL, prototypically to the mammillary bodies (138). Thus, as is well-established in the literature (139), the more anterior the lesion, the more severe is the confabulatory amnesia (140).

A separate, and important, issue, relates to GA's hunger. As discussed in the Results, there as a great paradox, in that the Brain Injury service had noted a *daily* concern for asking about hunger. It was remarkable that this issue *never* came up in the 56 psychotherapy sessions of GA's treatment. There are various possible explanations for this. At a concrete level it may be that therapy has no triggers for his hunger. So, for example, there are never the odors of food in therapy, he never sees other people eating, nor hears them talking about food. He also does not meet people who, in other settings, feed him and remind him of his hunger. A second explanation might be that “hunger” is a concrete manifestation of his more general emotional needs, and that these are more met in therapy, where he is being listened to. It is difficult to establish which of these is more likely. This it would be an interesting topic for future research. For example, what is his behavior like with people who are not *therapists*, but *are* also connected with triggers of food?

Categories of repetitive phenomena identified in the research with JL (47) were also present in the work with GA, but interestingly in a way that was specific to him. The reader is referred to the Results section, where each type of repetition occurring in GA's case is presented in detail.

### Recommendations

These patients are, at least in some respects, dramatically different from clients seen in conventional clinical practice. Thus, one might expect matters relating to psychoanalytical clinical technique to also different. However, it appears that the tried and tested fundamentals of psychoanalytic technique (107, 141) appear to apply just as much to therapeutic work with this group. Indeed, one might argue that the fundamentals are even *more* important with this amnesic population. Notably, core aspects of psychoanalytic techniques, such as holding, containment, reverie, free association, interpretation of unconscious content, and a non-directive and non-judgmental attitude in the therapist (141, 142), are *all* still relevant and applicable to the work with people who experience profound amnesia.

However, it may be that different types of psychotherapy (143, 144) are not equally appropriate for this group. These patients have profound impairments of recent memory, with consequences for other cognitive abilities, such as prospective memory. Thus, a therapeutic approach which place emphasis on non-cognitive, implicit memory abilities might well be more appropriate. We have previously discussed (47), an approach such as psychoanalysis seems well-placed to allow the patient to work around their cognitive impairments.

Memory loss also opens the ethical issue of the therapist possessing knowledge that the amnesic patient does not. This is especially important, given that the issue of “who holds knowledge” appears unprompted or spontaneously in the sessions. Is there a responsibility on the therapist to raise these issues with their amnesic patients? Or should amnesic patients expect the same level of psychoanalytic process in treatment as a non-neurological patients might? In other words, should therapists working with this population focus on, and respond to, the current material as it arises unprompted, and not introduce their own agenda and “steer” the treatment in directions informed by the therapist's (and the multi-disciplinary team's) sense of what is important.

Understandably, while this may be very important and urgent for the residential team and family members, it may *not* be the most important issue emotionally for the amnesic patient. Their lived experience, of the loss of temporal continuity and capacity to form new long term episodic memories, and the resultant emotional turmoil relating to this loss, may be the priority. From first principles, patients with amnesia deserve to have their psychological needs met in the order in which *they* prioritize them: the order in which they organically make their way into the therapy session. In that sense, then, we feel that one should adopt an approach similar to that of non-neurological patients in psychoanalytic treatment. Regardless, this poses ethical and technical issues that will face any psychoanalytically trained clinician seeking to work with this population, and is beyond the remit of this paper. However, we feel it is important to *begin* the discussion, on behalf of our neurological patients, and their clinicians.

A final and critical issue is the very *status* of psychotherapeutic work with profoundly amnesic patients. As discussed above, both JL and GA engaged effectively in an extensive and successful process of psychotherapy. They developed a working alliance, and were able to be reflective about the way in which they managed their feelings, and showed clear therapeutic gain. Remarkably, however, the existing literature suggests that such outcomes are unlikely, or perhaps impossible, or unimportant. Here, for example, is Blass and Carmelli's strong statement on the topic:

“We maintain that this biologistic perspective that underlies neuropsychoanalysis *runs counter to the essence of a psychoanalytic worldview*. While the proponents of neuropsychoanalysis argue that they are not reducing the psychological domain to the biological one… nevertheless, neuropsychoanalysis, in effect, ascribes to biology a kind of significance that does away with the value of meaning and psychic truth which is at the foundation of psychoanalysis”

Blass and Carmelli [(145), p. 35, *emphasis added*]

Thus, Blass and Carmelli appear to suggest that there is nothing to be gained from psychoanalytic work with neurological patients, and indeed that neuropsychoanalysis, as a field, is *in principle* irrelevant for psychoanalysis. As we have demonstrated above, this position seems hard to sustain in the light of the experience of JL and GA. The very existence of patients who are able to work psychoanalytically, despite losing an entire psychological skill, seems of enormous importance to psychoanalysis? To suggest that this “does away with the value of meaning and psychic truth” seems entirely unfounded, given that JL and GA's cases speak just as much to meaning and truth as do those of any other patient.

The very status of psychotherapeutic work is also addressed in a quite separate literature, this time from neuropsychologists working with neurological patients. In a survey of such clinicians, Judd and Wilson (146) report that memory impairment is the most substantial barrier to psychotherapeutic work:

“*…impaired memory is the most significant challenge. Here, continuity between sessions is hindered which, in turn, slows down the therapeutic process which may also obstruct the development of a working alliance.”*

*[Judd and Wilson* *(**146**), p. 443]*.

It is hard to imagine a more profound type of episodic memory impairment than that found in GA and JL, yet they plainly showed an ability to work in a therapeutic setting. However, it may be that a more Cognitive Behavioral Therapy (CBT) oriented approach (of the type surveyed by Judd and Wilson) would have been less successful with amnesic patients. One element of CBT, for example, is a requirement for patients to remember to “do their homework” between sessions, which relies on prospective memory skills that are absent in our patients. However, it may be that these authors have made (incorrect) assumptions about the role of episodic memory in psychotherapy? Our data suggest, of course, that recent episodic memory is *not* a central psychological skill for psychotherapy. Instead we suspect that other abilities are far more critical, of which emotion regulation is that with the best current evidence base (101, 102, 147–150).

## Conclusion

Single case studies, such as GA's, underline what *is*, and what is *not*, important about the psychotherapeutic process. On the one hand, such cases remind us of the *un*importance of recent episodic memory. Many authors appear to believe that memory is vitally important, or even critical, for psychotherapy. However, these cases remind us that this assumption may not be true. These remarkable individuals also remind us of the importance of the many well-established elements of psychoanalytic psychotherapy: the therapeutic alliance, the role of unconscious processes, holding and containment, reverie, transference dynamics, and so on. These are important elements of therapeutic technique for *all* clinical presentations, but seem especially relevant in these extreme cases of persons who present with a profoundly disabling brain injury, and yet are still able to make clinical gain.

## Data availability statement

The datasets presented in this article are not readily available because the data sets are audio recordings and transcriptions of live psychotherapy sessions containing sensitive and identifiable patient information. Requests to access the datasets should be directed to moorep1@tcd.ie.

## Ethics statement

The studies involving human participants were reviewed and approved by School of Human and Behavioural Sciences, Bangor University, North Wales. The patients/participants provided their written informed consent to participate in this study. Written informed consent was obtained from the participant for the publication of any potentially identifiable images or data included in this article.

## Author contributions

All authors listed have made a substantial, direct, and intellectual contribution to the work and approved it for publication.

## Conflict of interest

The authors declare that the research was conducted in the absence of any commercial or financial relationships that could be construed as a potential conflict of interest.

## Publisher's note

All claims expressed in this article are solely those of the authors and do not necessarily represent those of their affiliated organizations, or those of the publisher, the editors and the reviewers. Any product that may be evaluated in this article, or claim that may be made by its manufacturer, is not guaranteed or endorsed by the publisher.
