# Supplementary material for: Like a rolling stone: Psychotherapy without (episodic) memory
Source: Front Psychiatry. 2022 Nov 2;13:958194. doi: 10.3389/fpsyt.2022.958194 (PMC9666688; doi:10.3389/fpsyt.2022.958194)
Supplement: Supplementary file 1 [file Data_Sheet_1.docx]

Appendix A: Repetition

The outcome thematic coding conducted during previous research with JL (Moore et al., 2017) confirmed the presence of three clearly delineated and differentiated forms of repetition. Each form of repetition possessed its own specific phenomenology and functionality. An abridged description of these types of repetition is provided for the reader below:

1. *Regulatory Repetition*: the fixed and rigid repetition of information with little to no variation in terms of structure or content. This form of repetition was frequently observed following the abrupt interruption of the session’s temporal continuity. However, this type of repetition was also to be seen in moments of high emotional arousal. This suggests that repetition, in these instances, may not always be a consequence of organic brain injury but at times may have a defensive character. Irrespective of the causal factor it seems there is an unconscious mechanism, perhaps a prediction of sorts, which helps the person to retreat to safe ground when faced with the overpoweringly difficult feelings of confusion and fear of not knowing where he or she is or indeed what it was they were saying.

2) *Epistemological Repetition*: This is the repetition of explicit content which is variable and context dependent. This type of repetition was observed in relation to external situations experienced by the patient as puzzling. These contents were - amazingly, given the diagnosis of profound amnesia - brought repeatedly to therapy, it seems, in order to make sense out of confusion. Theoretically speaking, this form of repetition appeared to represent an epistomephillic drive, similar to those conceptualised by Freud, Klein and later Bion since it facilitated the transition from confusion to understanding, an allaying of anxiety associated with uncertainty, or from what might also be considered to be a part-object to whole-object mental representation. We believe this category of repetition is also a function of the transference-countertransference relationship between patient and therapist.

3) *Implicit Repetition*: This is the repetition of deep unconscious transference themes in the session content connected to material in the patient’s premorbid characterological structure. These implicit repetitions differ from the first and second types of repetition in terms of phenomenology and function. Phenomenologically they manifest as implicit underlying themes, contained in a wide range of spontaneously reported topics, not referred to explicitly in the content. Speculatively, the function of these implicit repetitions may serve to facilitate the expression of split off/projected, or yet to be fully integrated, very early internal object and object relationships as a means of relieving (re-living) and working through associated unconscious psychic tension. Such deep psychic phenomena while influencing present day dynamics and interactions do not constitute a response to them nor are they generated by them. Themes such as omnipotence, destructive impulses, hate, being cared for, self-care (or lack thereof), frustration, despair, envy, regret, and guilt are frequently to be seen in this form of repetition. Interestingly, while these unconscious aspects of mental functioning have been forgotten explicitly they are being expressed, repeated, and worked through implicitly and psychoanalytically in the therapy process.

Appendix B: Epistemological Repetition

Week 8

GA: when I was in college there were kids in my class who went to see shrinks, psychiatrists, psychologists, counsellors and all that – some of them had some really dark issues I think

PM: what’s it like for you coming here to see me for psychotherapy

GA: ah it’s ok I know there’s nothing seriously wrong with me mentally – I’m in good enough form most of the time it’s good to be able to come and talk about all the ups and downs

PM: would you like to say something more about them?

GA: well every now and then I can get sad – I miss home and I miss my mam – It’s hard being in Bellevue all the time. I only get to go home for a small bit at the weekend. Don’t get me wrong it’s great but it’s not long enough I’d like to stay at home for longer – it’s hard because when I go in the front door I am looking forward to meeting mam – and then my dad tells me that she has died. Contact with this difficult episode, amazingly retained by GA, immediately elicits an outpouring of profound grief. The grief appeared to be unmediated almost as if GA had just heard the news of his mother’s death. The crying and sobbing lasted for approximately two minutes and ended as suddenly as it erupted. GA reconnected with the therapist by enquiring about what the weather was going to be like at the weekend.

P: that must be so terribly hard for you

GA: it is .

GA then breaks down: there is a deep booming, harrowing, wail which seemed like raw unprocessed affect. The effect of which was to transport the therapist immediately to a mother who he had worked with clinically. The woman had experienced the death of two of her children. The therapist recalled that when the woman could grieve there was a similar raw affect present. An emotion that reverberates within your very being. Similarly GA’s grief had no soft edges. No gradual transition. Raw, visceral, ejected out of GA, irrupting into, and reverberating within the therapist. It the quality of an infantile mood where the baby can flip in and out of emotional states rapidly.

After approx. 2 mins crying and some silence

GA: Do you know what the weather is going to be like the weekend Paul?

PM: It’s supposed to be good G – I’m wondering about what just happened?

GA: what happened?

PM: We were talking about your mam, and you were really upset… (starts to cry again but not so strongly) you know it’s strange, like I know she’s gone I know that sitting here talking to you but it’s like I forget when I walk through the door at home … I still expect to see her.

Week 26:

GA: How long have I been coming here now Paul?

PM: Could you hazard a guess?

GA: Two years is it?

PM: Not quite that long – a little over 6 months

GA: When did we start?

PM: October

GA: I like coming here – I love the couch, and I get to talk about whatever is troubling me

PM: And is there anything troubling you in particular at the moment G?

GA: I’ve been thinking about my mam a lot and I really miss her - I can’t believe she’s gone she was so young… (Breaks down uncontrollably) …

PM: It’s really really painful for you

GA: It is Paul... It’s like the first day I found out she had died.

PM: Do you remember that?

GA: not really I’m told I was in the hospital when I found it but I don’t remember that’s the story I’ve been told… I can’t make sense of it.. I still expect to see her when I go home and that’s really tough … it’s tough on my dad too because he has to remind me.

Week 48:

During a conversation about GA’s university studies:

GA: I really want to be able to get my memory good enough to be able to continue my studies… Mam would have wanted me to do that … she really valued education and loved learning new things herself… It’s such a shame she was taken so young… (cries but not the booming cry that used to be there).

PM: what’s that like for you now?

GA: ah it’s still hard you know but it’s getting easier .. I still miss her when I go home but I don’t expect to see her as much… and that’s a lot easier for my dad.

Appendix C: Countertransference

*I find myself thinking a lot about what does the weather mean in our sessions? I eventually come to the conclusion that he is trying to forecast what the emotional climate will be like at home when he gets there – how will dad be?*

*At times G’s questions can come fast and furious and I can find myself feeling under pressure to keep up – my head is swirling.*

*Sometimes he would ask me do I remember something he told me before for example what kind of car did his school friend drive? Even though he has told me this – must be hundreds of times in the moment I couldn’t retrieve it from my memory (I subsequently realise, upon reviewing the sessions for research, this is because GA confabulated slightly and the models of cars could vary in his retelling of the story).*

*When he touches on his mam’s death he can very quickly go very deep into it and become very very upset a really profound and what seems like primal heart wrenching grief a booming sobbing kind of crying – it is profoundly sad – which can end as abruptly as it begins this is very difficult to deal with as an analyst as he moves on very quickly and I am left holding the grief and feeling very sad – while he is talking about sporting achievements – a very quick change of gears emotionally one that can find tough to keep up with*.

*At times some of G’s questions can just irrupt into the session they seem to come out of nowhere or at least outside the current context of the session, for example out of the blue he asked me about my job title in these moments I can find myself wrong footed and in a state of confusion trying to adjust to the change of narrative/ topic/ pace/ relational depth it can be hard to orient myself and difficult to gather my mind – this can happen with non-neurological patients too but there is something about the abruptness of this with G that is different more severe more jarring.*

*GA speaks about a school acquaintance EB who I have come to understand as a repository of dark and difficult emotions – he speaks about EB’s alcohol and drug use and I have a very strong visceral reaction to this character when he enters the therapy – one of stomach churning anxiety – as this particularly narrative develops I find myself struggling to ground myself and thinking about the health care assistant who brought him to the appointment and how I had in the interaction at the door picked up a really bad vibe off the person who seemed to be annoyed and cross, the person was new and I needed to explain to her that we would be finished at a certain time and when to come back for GA – it was as if the HCA didn’t take kindly to me telling them what to do and resented me for it – I then find my mind turning to wondering was this why GA seemed to be noticeably anxious at the beginning of the session – and why EB came into the session - he seemed to be sad and anxious, and was complaining about the residential unit (which was not typical) the session continues and EB material contains themes of self-harm and destructiveness and then oscillates to the glorious excess of something or other one of the narrative screens (repetitive scripts) are wheeled in, in what I now believe to be a tried and tested method to restore affective balance to GA’s mind – I’m left wondering what is going on here? is he attempting to emotionally regulate himself? is he upset about something? Is it the person who has brought him here today? Has something happened? If it had G wouldn’t be able to remember explicitly and relate this to me. Not long after in the session, G brings his Mam up and opens up some more to me about missing her.*

*Definitely sems to be an improvement in the transference relationship seems able to ask me more direct questions about myself and the space although I see him doing his with his assistant today?? And it makes me wonder is it defensive i.e. if I ask the question I won’t be ask questions! He already told me that his fine most of the time except when he is asked questions – a lot more today about the negative side of his emotional world was able to tell me that he feels disappointed and frustrated when he can’t go home for the weekend and the fact that he wishes he is at home.*

*I feel a lot more anxious today with GA not sure what that is about – globally, a bit of terror might be linked to the fact that we are moving deeper into the work now and there is a greater sense of uselessness powerlessness particularly around the repetition and the going on about the same old stuff while there is the sense of frustration at the same old story coming up there is also a feeling of horror/terror/ fright at the back of it a subtle distinction between the different repetitive mechanisms seems to be materialising. There are the conversational repetitions where the topics generally converge on these repetitive theme of the aunt’s shops or basketball or the institute or there also seems to be a defensive cutting off from high emotion and then a reconnect through the weather or through the time or amount of sessions we have had – while he can’t’ remember how many times we have met he does remember that it is open ended he also remembered my full name without prompting this is ten sessions in how does this happen? Is it now a semantic memory I am confused and trying to understand is this purely an episodic amnesia ? there was also the mention of girlfriends today the MB and then later RA who plays the same sport as GA lots in common and still friendly when GA meets her … moving piece about his mam interestingly his cry is very like his laugh it is instantaneous and free has the strange feeling of being unmediated – raw affect – it is booming and unconstrained emerges very quickly and powerfully and subsides just as quickly as it came –first time he has cried here it is terribly sad to think that the only time he realises that his mam has passed away is when he is coming home on Friday the realisation is tagged to that procedural memory.*

*Started off with what my job title is … I wonder because it came up towards the end in a kind of fantasy confabulation piece about what therapy might mean for GA it is as if he feels he might be being evaluated as part of the service and if he has a bad session he may not be allowed to go home he has no idea if this has ever happened or not but the idea is/was taken that he must be good and he must perform well or it could impact upon his freedom and accessing the thing he most likes - being at home – this would explain his over eagerness to apologise for the least little thing – I wonder is this a feature of all Brain injury? Amnesic patients in residential settings – they feel totally dependent upon / at the mercy of the services – must explore this with GA next time it comes up, and in doing so re-emphasise the separateness of the therapy this is also a paranoid piece – something incredibly sad about that – not knowing how long he has been there all that time not knowing his mam has passed or the details of it today his grandmothers came into it and they are both incredibly resilience and strong women.*

*Strange one today, in so far that I am quiet and finding it difficult to be present . I wonder does this signify a development of sorts some resistance to the process as while if I remember correctly he did start off with the school story and the way they’re going to see psychiatrist, psychologists, shrinks and a voodoo doctor this came up again later too so it might be ???*

*GA seemed very tired and very sad at times like he was fed up and didn’t really want to be here – some of his material had threads and other possible currents it seems as if the scripts are wheeled out as a means of holding maintaining containment for G then some new material will come in one such piece is the introduction of the rich kids only saw their dads (not mothers) once a month and everything else was provided by care staff and coordinators – obvious link here to G’s own living arrangement. When I pointed this out he became defensive and said correctly I see my dad every week - I am wondering now whether he does actually get to see his dad every week?*
